# Noncontact and Wide-Field Characterization of the Absorption and Scattering Properties of Apple Fruit Using Spatial-Frequency Domain Imaging

**DOI:** 10.1038/srep37920

**Published:** 2016-12-02

**Authors:** Dong Hu, Xiaping Fu, Xueming He, Yibin Ying

**Affiliations:** 1 College of Biosystems Engineering and Food Science, Zhejiang University, 866 Yuhangtang Road, Hangzhou, 310058, China; 2 Faculty of Mechanical Engineering and Automation, Zhejiang Sci-Tech University, 928 Second Avenue, Xiasha Higher Education Zone, Hangzhou, 310018, China

## Abstract

Spatial-frequency domain imaging (SFDI), as a noncontact, low-cost and wide-field optical imaging technique, offers great potential for agro-product safety and quality assessment through optical absorption (μ_a_) and scattering (μ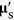) property measurements. In this study, a laboratory-based SFDI system was constructed and developed for optical property measurement of fruits and vegetables. The system utilized a digital light projector to generate structured, periodic light patterns and illuminate test samples. The diffuse reflected light was captured by a charge coupled device (CCD) camera with the resolution of 1280 × 960 pixels. Three wavelengths (460, 527, and 630 nm) were selected for image acquisition using bandpass filters in the system. The μ_a_ and μ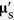 were calculated in a region of interest (ROI, 200 × 300 pixels) via nonlinear least-square fitting. Performance of the system was demonstrated through optical property measurement of ‘Redstar’ apples. Results showed that the system was able to acquire spatial-frequency domain images for demodulation and calculation of the μ_a_ and μ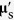. The calculated μ_a_ of apple tissue experiencing internal browning (IB) were much higher than healthy apple tissue, indicating that the SFDI technique had potential for IB tissue characterization.

Recently, optical characterization of agro-products has been increasingly explored for its great potential in food safety and quality inspection^1^,^2^. Although considerable research and much progress has been reported for visible and near-infrared (Vis-NIR) spectroscopy for food safety and quality detection over the last two decades^3^,^4^, the Vis-NIR measurement approximately describes the aggregate effect of light interaction with biological tissues, and cannot decouple light absorption from scattering. Hence the conventional Vis-NIR measurements are not suitable for quantitative analysis of light absorption and scattering in scattering-dominant agro-products.

Light absorption is related to tissue chemical constituents, such as moisture and soluble solids, while light scattering provides physical and structural information, such as firmness, elastic modulus, and cell size^5^. The optical properties can be characterized by an absorption coefficient (μ_*a*_) and reduced scattering coefficient (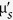). The time-resolved method^6^, frequency domain method^7^, spatially resolved method^8^, and integrating sphere method^9^, have been reported for recovering optical properties of biological tissue. However, expensive instrumentation for time-resolved and frequency domain methods hinders their applicability on agro-products. In addition, these methods use single-point-source illumination systems to measure the tissue optical properties, which is insufficient in characterizing the spatial variability and optical properties of agro-products composed of homogeneous or heterogeneous layers^10^.

As a noncontact, low-cost and wide-field imaging technique, structured illumination coupled with a light transfer model can be utilized to quantitatively measure tissue optical absorption and scattering coefficients on a pixel-by-pixel basis. This technique is known as spatial-frequency domain imaging (SFDI). Compared with other measurement methods (i.e., time-resolved, frequency domain, spatially resolved and integrating sphere methods), the SFDI technique has the unique capability of quantitatively decoupling light absorption from scattering in a wide field-of-view. The optical imaging technique was first proposed and applied successfully by Cuccia *et al*.^11^,^12^, and it has been widely applied in the biomedical field over the last decade. Differences between the optical properties of normal and abnormal tissues obtained through the SFDI technique may be used to identify diseases in biological tissue. For instance, the μ_*a*_ was shown to be generally lower in Alzheimer patients’ brains, while the 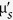 was much higher^13^. Burn wound infections had relatively higher μ_*a*_ and lower 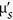, and could be distinguished from non-infected burns^14^. Burn wound severities in a porcine model were assessed accurately and quickly after injury through monitoring changes in 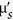 and blood flow^15^. In addition, changes in tissue chemical constituents (i.e., stO_2_, oxygen, water, hemoglobin) obtained by the SFDI technique were also used as indicators for diagnosing diseases such as port wine stain^16^ and breast pathology^17^. Despite its success in the biomedical field, SFDI is far from being exploited for nondestructive sensing of agro-products. To our knowledge, its application on agro-products was limited to bruise detection studies on apples reported by Anderson *et al*.^10^ and Lu *et al*.^18^.

A low-cost SFDI system operated in several wavelengths was constructed and developed in our laboratory. The wavelengths could be altered through selection of bandpass filters, and three wavelengths in the visible spectrum (460, 527, and 630 nm) were used in this study. The system was capable of acquiring spatial-frequency domain images in a large field-of-view (192 × 144 mm^2^). The SFDI instrumentation was described in detail and tested using a tissue-simulating solid optical phantom. Optical property image maps of ‘Redstar’ apples were then measured to demonstrate overall performance of the system. The objectives of this research were to:

- Present details of the SFDI system, including system components, software, operation and calibration.
- Evaluate whether the SFDI system was capable of μ_*a*_ and 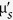 measurement on a pixel-by-pixel basis.
- Demonstrate performance of the SFDI system on an agro-product, i.e., the μ_*a*_ and 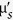 measurement and internal browning (IB) tissue characterization in ‘Redstar’ apples.

## Results

### System Calibration Results

SFDI system calibration was performed through five measurements of the μ_*a*_ and 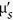 of a solid optical phantom. The results were summarized (Table 1) and compared with true characterization reports at each wavelength (460, 527, and 630 nm) provided by the INO. The measured relative error was generally higher in μ_*a*_ than 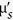. Since the solid optical phantom was a scattering-dominant material (μ_*a*_ ≪ 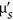), this error was expected in the SFDI measurement results.

### Optical Properties of ‘Redstar’ Apples

**Figure 1:**
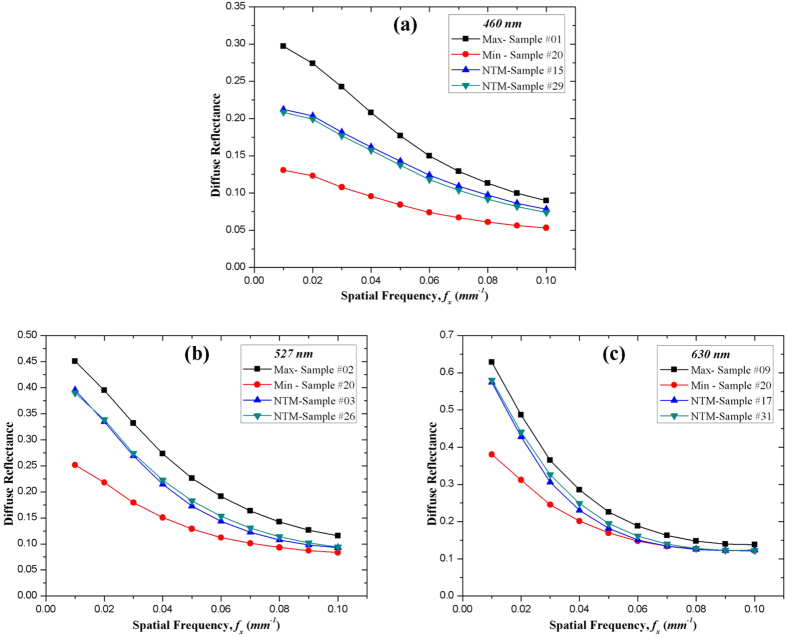
Diffuse reflectance of apple samples vs spatial frequency of illumination for measured surface #2 at 460, 527 and 630 nm recorded by the SFDI system. NTM indicates the apple samples nearer to mean values of the diffuse reflectance.

In Fig. 1, the relationship between the average diffuse reflectance of measured apple surface #2 and the spatial frequency of illumination at 460, 527 and 630 nm are presented. The maximum, minimum curve and another two curves close to the mean values of diffuse reflectance were selected as representatives among the 36 acquired sample curves. The results consistently demonstrated that the diffuse reflectance decreased when the spatial frequency increased, with dramatic drops between the lower frequencies and a relatively slower decline at high frequencies. The high frequency, structured illumination patterns used contained larger regions of low light intensity than lower spatial frequencies. Therefore, the reduction in diffuse reflection intensity observed was reasonable. Moreover, the diffuse reflectance at 630 nm was the highest, followed by 527 nm and 460 nm in order. Differences in reflected wavelength intensity was due to absorption properties of the apple tissue. Sample #20, with the minimum reflectance, showed the same trend. This trend was consistently observed in apple results, demonstrating that the μ_*a*_ at 460 nm was the highest. A 15–20% variation in diffuse reflectance was observed at 460 nm, which may have been caused by differences between individual apple samples. Similarly, the diffuse reflectance of measured surface #1 and #3 present the same variation trend.

**Figure 2:**
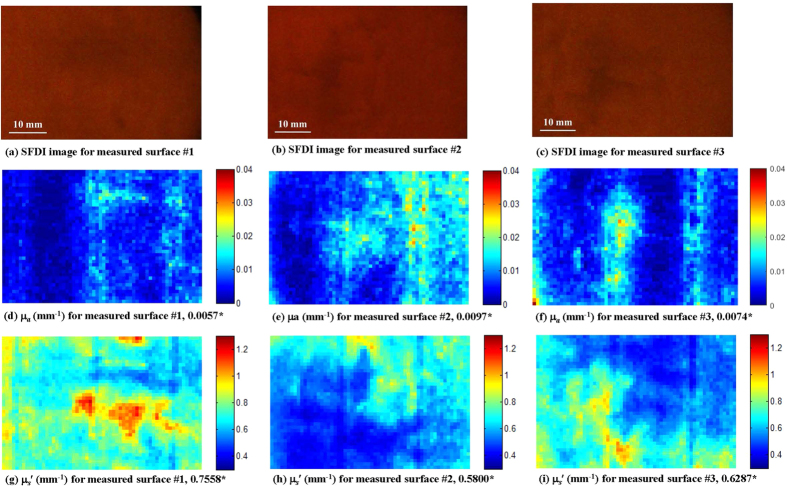
Optical absorption and scattering image maps (mm^−1^) of sample #3 at 630 nm. **(a), (b), (c)** SFDI images for measured surface #1, #2, #3; **(d), (e), (f)** optical absorption image maps for measured surface #1, #2, #3; **(g), (h), (i)** optical scattering image maps for measured surface #1, #2, #3. Asterisks (*) indicate average optical properties in the ROI.

**Figure 3:**
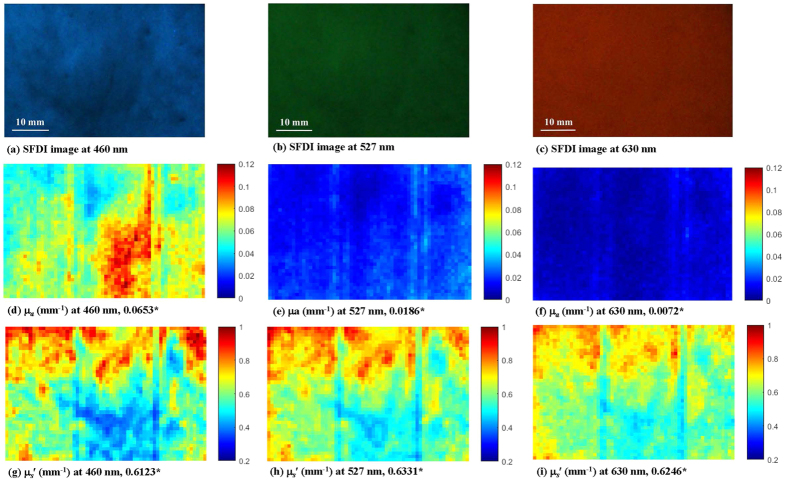
Optical absorption and scattering image maps (mm^−1^) of sample #2 for measured surface #2. (**a**), (**b**), (**c**) SFDI images at 460, 527, and 630 nm; (**d**), (**e**), (**f**) optical absorption image maps at 460, 527, and 630 nm; (**g**), (**h**), (**i**) optical scattering image maps at 460, 527, and 630 nm. Asterisks (*) indicate average optical properties in the ROI.

In order to explore the optical properties at different locations of a fruit, the μ_*a*_ and 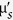 image maps of sample #3 at 630 nm were plotted in Fig. 2. The 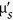 of sample #3 was closest to the average 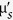 value of the 36 samples. The figure displays that the 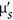 was much higher than the μ_*a*_, for example, 0.5800 mm^−1^ ≫ 0.0097 mm^−1^ for measured surface #2, which indicated that the apple tissue was scattering dominant. It was known that tissue optical properties were independent of thickness in terms of diffusion approximation. Figure 2 shows discrepancies among the optical properties calculated from the three measured apple surfaces. Although the average thickness of part A was 25 mm, the apple tissue was thinner at the edges than in the center, which may have influenced optical property estimation. The apple samples were sliced manually therefore, differences in sample handling may have potentially affected the optical property measurements. Moreover, the three measured surfaces were made on different parts of the apples, making this issue more complicated as tissue thickness may not be the only factor causing the discrepancies. The structural properties and chemical constituents may vary from the apple peel to the core, adding more challenges to the measurement. Figure 3 displays the representative μ_*a*_ and 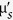 image maps at 460, 527, and 630 nm of a single apple measured surface of sample #2. Sample #2 had a μ_*a*_ value closest to the average value of the 36 samples. The three wavelengths used in the experiment had different light absorption, scattering and transmission abilities, which resulted in fluctuations in μ_*a*_ and 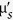 for the same pixel or ROI. Compared with the optical properties calculated at 527 and 630 nm, the μ_*a*_ at 460 nm was much higher. Measured surfaces #1 and #3 also presented this phenomenon. 460 nm was near an absorption peak corresponding to a combination of chlorophyll-a and carotenoid constituents in the apple tissue, and the μ_*a*_ was about 5–10 times greater than the other wavelengths^19^,^20^. Conversely, the 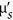 at 460 nm was much lower than those at 527 and 630 nm due to the chlorophyll-a and carotenoid absorption peaks^20^. The 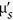 at 527 and 630 nm did not follow the behavior strictly explained by the Mie theory, where a steady decrease in 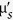 as the wavelength increased was expected. Inhomogeneity of the apple tissue may be one of the factors which contributed to this phenomenon since the Mie theory is applicable towards homogeneous tissues. In the optical phantom, which was regarded as an ideal optical medium, the 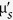 decreased as the wavelengths increased (Table 1). Previous studies^5^,^20^ indicated that the 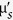 decreased with increasing wavelengths in 500–1000 nm, with about 15% relative variation. It was reported that a simple wavelength-dependent function (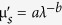, where *a* and *b* are parameters for the power series model) was frequently used for a second curve fitting to obtain a smooth spectrum of 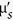, which would follow the behavior of Mie theory^21^.

### Internal Browning Tissue Characterization

**Figure 4:**
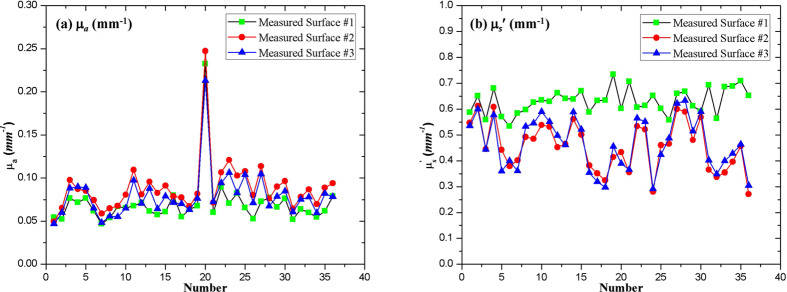
Optical properties of 36 apple samples for measured surfaces #1, #2, and #3 at 460 nm.

The μ_*a*_ and 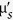 of the 36 apple sample ROIs (200 × 300 pixels) were averaged, and their values at 460 nm were plotted and presented in Fig. 4. The discrepancies between the optical properties of the three measured surfaces may have been caused by tissue inhomogeneity, relatively thinner tissue at the edge of part A, and differences in sample handling during slicing, and thus, it could not be treated as thickness issue alone. In Fig. 4(a), outlying points were recorded for sample #20, peaking at 0.2326 mm^−1^, 0.2474 mm^−1^, and 0.2130 mm^−1^ for measured surfaces #1, #2, and #3 respectively. The average μ_*a*_ of the other 35 apple samples were 0.0656 ± 0.0099 mm^−1^, 0.0841 ± 0.0162 mm^−1^ and 0.0755 ± 0.0152 mm^−1^. The 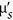 of sample #20 (0.6025 mm^−1^, 0.4333 mm^−1^, and 0.3889 mm^−1^) were close to the others (0.06318 ± 0.0480 mm^−1^, 0.4564 ± 0.0929 mm^−1^, and 0.4679 ± 0.1009 mm^−1^ for measured surfaces #1, #2, and #3 respectively).

**Figure 5:**
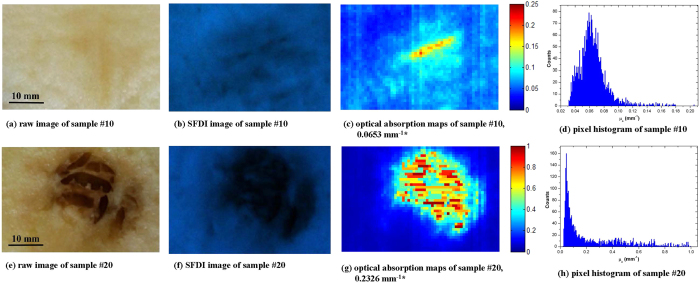
SFDI results of healthy (sample #10) and IB (sample #20) apple tissue for measured surface #2 at 460 nm. (**a**), (**e**) and (**b**), (**f**) raw and SFDI images in the ROI respectively; (**c**), (**g**) quantitative μ_*a*_ image maps; (**d**), (**h**) corresponding pixel histograms. Asterisks (*) indicate average μ_*a*_ in the ROI.

The sample #20 was further studied to attempt to explain why the μ_*a*_ was unusually large. As shown in Fig. 5, internal browning was occurring within the sample. SFDI results of sample #10, which were similar to the other 35 healthy apples, were used for comparison with sample #20. In Fig. 5(e) and (f), we can see the IB in the sample #20 clearly. In Fig. 5(g), the μ_*a*_ in the healthy regions ranged 0.0246 mm^−1^ to 0.2000 mm^−1^, while the values in the IB region were much higher than 0.2000 mm^−1^, with the maximum value peaking at 0.995 mm^−1^. The wide range observed in the μ_*a*_ (0.2000 mm^−1^ to 0.995 mm^−1^) of the IB region may be due to tissue inhomogeneity, which could also be observed in Fig. 5(e). Moreover, the IB tissues were discontinuous and contained many cavities filled with air, which weakened the tissue scattering effects, resulting in higher μ_*a*_ for the IB region. Distribution of the μ_*a*_ may be more uniform if the IB tissues were more homogeneous. Vanoli *et al*.^22^ reported that the μ_*a*_ was higher in IB ‘Braeburn’ apples than in healthy ones, and it significantly increased with IB severity. Those results are consistent with the results interpreted in this study. Anderson *et al*.^10^ reported that there was a lower 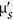 in the bruised apple regions than the non-bruised ones from 650 to 980 nm, while the μ_*a*_ did not show obvious or regular changes. Bruising is an external or subsurface defect, which is caused by impact or mechanical damage during harvest, transport or handling^23^,^24^, while IB is an internal disorder without external symptoms, resulting in unsightly browning and cavities in affected flesh^25^. Due to the discrepancies between the chemical constituents and physical structures during emergence of the two different defects, the measured optical properties presented different trends. Wavelength selection may be another factor causing differences between these two studies. Therefore, the SFDI technique should be applied to study the variation in the calculated μ_*a*_ and 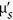 in IB apple tissues and their relationship with structural properties and chemical constituents in future research.

The average μ_*a*_ and 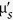 of ‘Redstar’ apples for the three measured surfaces at 460, 527, and 630 nm are summarized in Table 2. The results of healthy apples were averaged from 35 samples. Compared with the results reported by other researchers using different techniques, the μ_*a*_ and 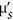 obtained in this study were similar. Lu *et al*.^23^ reported that the μ_*a*_ and 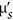 of ‘Golden Delicious’ and ‘Red Delicious’ apples between 500 and 1000 nm were 0.01–0.09 mm^−1^ and 0.75–0.9 mm^−1^ measured by a hyperspectral imaging-based spatially resolved technique. Rowe *et al*.^20^ used an integrating sphere method to measure the optical properties of ‘Royal Gala’ apples from 400 to 1050 nm, and the results were 0.01–0.15 mm^−1^ and 0.8–1.6 mm^−1^ for μ_*a*_ and 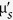 respectively. Due to differences in apple cultivar, wavelength range, data acquisition method, instrument calibration, and inverse algorithm implementation, differences in reported μ_*a*_ and 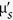 were expected. Additionally, tissue complexity, inhomogeneity and diversity among individual apple samples may also affect reported results.

## Discussion

Spatial-frequency domain imaging is a novel technique in the agricultural field which could quantitatively obtain optical absorption and scattering properties in diffuse media. In this study, an SFDI system was developed for the measurement of μ_*a*_ and 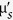 in apple fruit. The system was able to acquire spatial-frequency domain images and calculated the μ_*a*_ and 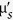 at certain wavelengths (i.e., 460, 527 and 630 nm) in the visible spectral regime. The wavelengths in the SFDI system could be altered using various bandpass filters, thus making the system flexible. The system was applied to ‘Redstar’ apples to demonstrate that the SFDI technique was suitable for the optical property measurement in a wide field-of-view and was promising for internal browning tissue characterization. Compared to other methods of optical property measurement, the SFDI technique showed great potential for noncontact, quantitative measurement on a pixel-by-pixel basis, and provided spatially varied information on the μ_*a*_ and 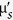. High spatial frequency illumination patterns provided more information pertaining to the surface tissue, while the low spatial frequencies penetrated deeply into the tissue with higher depth sensitivity^18^, making this technique promising for optical property measurement of multi-layered tissues.

The SFDI technique in agricultural field is at an early stage and this study was implemented completely in a laboratory setting. Manual slicing of the samples may damage or even change tissue structure or composition, which added a considerable challenge to optical property measurement and the implications of this issue has yet to be considered in research. Soluble solids content (SSC), for example, related to the apple maturity, might be affected during manual slicing and then contributed to the optical properties, especially for the μ_*a*_. The microstructural characteristics of apple tissues were related to the 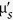 and might also be destructed from the slicing, which would further affect the measurement^5^. The relation among the optical properties, chemical constituents (e.g., SSC) and structural properties was expected to be studied in our further research. Therefore, future research should consider the creation of standard sample slicing procedure to improve the measurement accuracy. Moreover, attention should be paid to improve the light source in the NIR spectral regime since it could potentially provide more tissue information related to agro-product quality (i.e., soluble solids content, acidity, firmness)^26^. Furthermore, more sophisticated modeling methods with curved surface correction of agro-products is in great demand, which is the basis of noninvasive measurement. Aiming for defect detection in intact agro-products in the future, it is critical that improvements to the algorithm and overall speed of taking measurements and calculations are made (e.g., reduction of frequencies and/or phases). It is expected that this system will find more practical applications (qualitative and/or quantitative) in the area of agro-products safety and quality inspection by measuring the absorption and scattering properties.

## Methods

### Light Transfer Models for Determining Tissue Optical Properties

Different light transfer models have been developed for describing light propagation in turbid biological tissues and determining intrinsic optical properties. Among them is the diffusion approximation, which has been demonstrated in combination with structured illumination in the spatial-frequency domain imaging technique^12^. The SFDI technique uses spatially modulated structured illumination patterns projected onto a sample in the form:


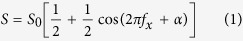


where *S*_0_, *f*_*x*_ and *α* are the illumination source intensity, spatial frequency (mm^−1^), and spatial phase respectively. The diffuse reflected light intensity *I*(*x*, *f*_*x*_), is captured from the sample by a charge coupled device (CCD) camera:


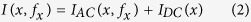


where *I*_*AC*_(*x*, *f*_*x*_) and *I*_*DC*_(*x*) are the measured *AC* and *DC* components of the diffuse reflected light intensity respectively. *I*_*DC*_(*x*) is a function of spatial location *x* and it is constant in the same spatial frequency *f*_*x*_. *I*_*AC*_(*x*, *f*_*x*_) is a function of *x* and *f*_*x*_, and it can be characterized as:


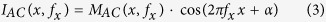


where *M*_*AC*_(*x*, *f*_*x*_) is the amplitude envelope of the diffusively reflected photon density.

In order to obtain *M*_*AC*_(*x*, *f*_*x*_), the sample should be illuminated with a sinusoidal pattern three separate times per spatial frequency at three different phase offsets, *α* = 0, *α* = 2*π*/3 and *α* = 4*π*/3 radians^27^. Then *M*_*AC*_(*x*, *f*_*x*_) can be calculated using the demodulation expression:


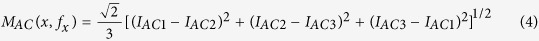


where *I*_*AC*1_, *I*_*AC*2_ and *I*_*AC*3_ are the *I*_*AC*_ image values at each spatial location with shifted spatial phases. According to equation (2), it is deduced that *I*_*AC*_(*x*, *f*_*x*_) = *I*(*x*, *f*_*x*_) − *I*_*DC*_(*x*), and we substitute it into equation (4):


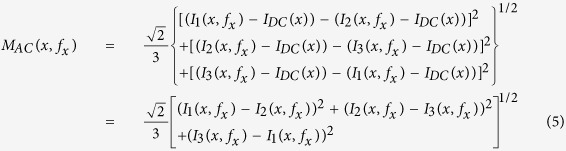


Diffuse reflectance of the sample *R*_*d*_(*x*, *f*_*x*_) can be measured using a reference calibration plate with following equation:


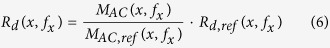


where *M*_*AC,ref*_ (*x*, *f*_*x*_) presents the amplitude envelope of diffuse reflected light of the reference calibration plate, *R*_*d, ref*_(*x*, *f*_*x*_) is the diffuse reflectance of the reference calibration plate. The μ_*a*_ and 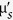 can be obtained via nonlinear least-square fitting by following equation:


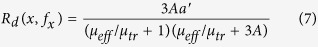


where 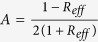 is a constant, 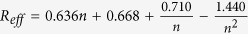, *n* is refractive index of the sample, 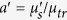 is the reduced albedo, 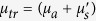 is the transport coefficient, and *μ*_*eff*_ = (3*μ*_*a*_*μ*_*tr*_)^1/2^ is the effective attenuation coefficient. To decouple absorption from scattering by equation (7), a minimum of two spatial frequencies with three spatial phases are needed theoretically.

### System Establishment and Software

**Figure 6:**
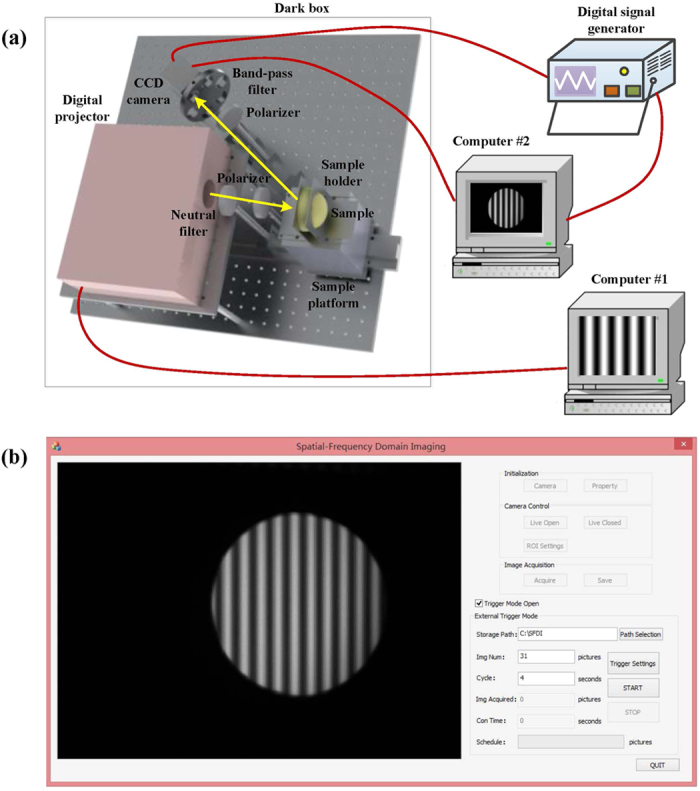
SFDI system schematic and software. **(a)** Schematic diagram of the SFDI system**; (b)** Visual Studio interface software for image acquisition and system control.

A schematic diagram of the SFDI system is illustrated in Fig. 6(a). The system was constructed on an optical breadboard and operated in a dark box with dimensions of 600 × 800 × 1200 mm^3^ to avoid the influence of ambient light. A simple digital projector-based digital light processing (DLP) light engine (NEC V260W+), utilized an ultra high performance (UHP) mercury lamp to illuminate the test samples. To create the sinusoidal illumination patterns, grayscale bitmap images were generated using MATLAB (The Mathworks Inc.). The images were loaded into a PowerPoint presentation file in a certain order and automatically played. The projection of the patterns was periodically controlled by computer #1. The structured light passed through a neutral filter (NE2R05A, Thorlabs Inc.) placed in front of the DLP lens to reduce light intensity uniformly. The test sample was placed on a height-adjustable platform to guarantee that the structured light reached the center of the sample. Sample holders were designed to suit different thicknesses of agro-products with a range of 0–118 mm. The reflected light was captured by a frame-transfer CCD camera (DVP 30GC03E) capable of imaging up to 30 frames/second at full 1280 × 960 pixel resolution. The camera was directly connected to computer #2 via USB, through which both camera control and data transfer were operated. A bandpass filter wheel was placed in front of the CCD camera, allowing for use of interference filters for detection of a narrow wavelength band. Three different bandpass filters in the visible spectral regime, with wavelengths of 460, 527 and 630 nm and bandwidth of 10 nm (BP 460/20k, BP 527/10k, and BP 630/10k) were used in this study. Additionally, two crossed linear polarizers were added in the illumination and detection arms to avoid specular reflection from the sample surface. All the optical components in the SFDI system were mounted on two digital scale, dovetail guide rails to make distance adjustments simple and accurate. A digital signal generator was used to trigger the CCD camera for periodic image acquisition, and was connected to computer #2 using a serial port line. The luminance nonlinearity of the digital projector may lead to distortion of the illumination pattern, making it non-sinusoidal. Several steps were taken to linearize projection: i) the grayscale bitmap images were projected onto a standard Teflon plate; ii) the average pixel intensity from the camera was recorded and normalized to the brightest projection; iii) using the projector response function as a look-up table, the input image signals were adjusted to make the detected projected image sinusoidal^28^. In this study, image acquisition cycle was set to be four seconds, and 10 spatial frequencies with three phases were selected, generating a total of 30 grayscale bitmap images per sample. In total, including reference image acquisition time, it took 124 seconds (31 × 4) to complete one measurement. In most SFDI studies, spatial frequencies below 0.3 mm^−1^ were used^29^. It was reported that multi-frequency fits could provide a more stable and accurate measurement of the average optical properties^12^. However, too many frequencies were not practical in measurement as the fitting would be time-consuming. Considering both measurement accuracy and fitting speed, 10 spatial frequencies were chosen between 0.01 mm^−1^ and 0.1 mm^−1^ at an interval of 0.01 mm^−1^ in this study. Zero frequency could provide the most absorption contrast and the best signal-to-noise ratio, however, it was challenging to implement surface height or angle curvature correction^30^. Therefore, the zero frequency was excluded at the cost of the decrease in absorption contrast in this study.

The parameterization and image acquisition interface software for the SFDI system was developed on a platform of Visual Studio 2012 and Open Computer Vision in the Microsoft Windows operating system. Software Development Kits (SDK) provided by the manufacturers of the CCD camera were used in the Visual Studio programming environment to satisfy various functions such as image acquisition, system control, and synchronization. The system software was able to fulfill both single image acquisition and periodic acquisition automatically. Automatic image acquisition by the CCD camera could be fulfilled by an external triggering of the digital signal generator controlled by the computer #2 using a serial port line. Storage path, image number and acquisition cycle were set before experiment, and the software could display image acquisition schedule on a real-time scale, such as consumed time and acquired image number.

### System Operation

The SFDI measurement process consisted of the following steps. (i) The digital projector was warmed up for ten minutes to make the output illumination as uniform as possible. Exposure time and gain of the CCD camera were adjusted according to the wavelength and test sample in order to obtain a higher signal-to-noise ratio. The external trigger mode of the digital signal generator was set to allow the CCD camera to capture images automatically and periodically, which was synchronized with the PowerPoint presentation file. (ii) The reference calibration plate was mounted on the platform using the sample holders for image acquisition. After the measurement was completed, the acquisition wavelength was altered by rotating the bandpass filter wheel and the next SFDI measurement was made. (iii) The reference calibration plate is replaced with a test sample and the measurements at all wavelengths were carried out in sequence. (iv) A dark image was acquired with the digital projector turned off. Data processing, including selection of ROI, demodulation and nonlinear fitting, were carried out afterward.

### Sample Preparation

The laboratory SFDI system was calibrated using a solid optical phantom with known optical coefficients. The phantom was used as a reference material provided by INSTITUT NATIONAL D’OPTIQUE (INO) in Quebec city, Quebec, Canada^31^. The model number of the optical phantom was PB0302 with overall dimensions of 85 × 80 × 10 mm^3^. The penetration depths using planar light at 460, 527, and 630 nm, were approximately calculated using the function^1^ (
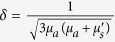
) based on the given optical properties, and were all less than 10 mm (7.6 mm, 8.6 mm, and 9.6 mm respectively). Monte Carlo simulation results showed that the relative errors of diffuse reflectance of the 10 mm-thick phantom were 2.95%, 3.73%, and 4.18% at the three wavelengths selected, compared to that of a 40 mm-thick phantom. Moreover, the average values of relative standard deviations in the region of 0–15 mm were all less than 3%, which demonstrated that the phantom was adequately thick as a calibration material. It should be noticed that thicker phantoms are recommended to minimize edge effects, especially for those with low optical properties^32^. Titanium oxide (TiO_2_) was used as a scattering agent, while carbon black was used as a universal absorbing dye, allowing for long term stability and reproducible optical property phantoms.

**Figure 7:**
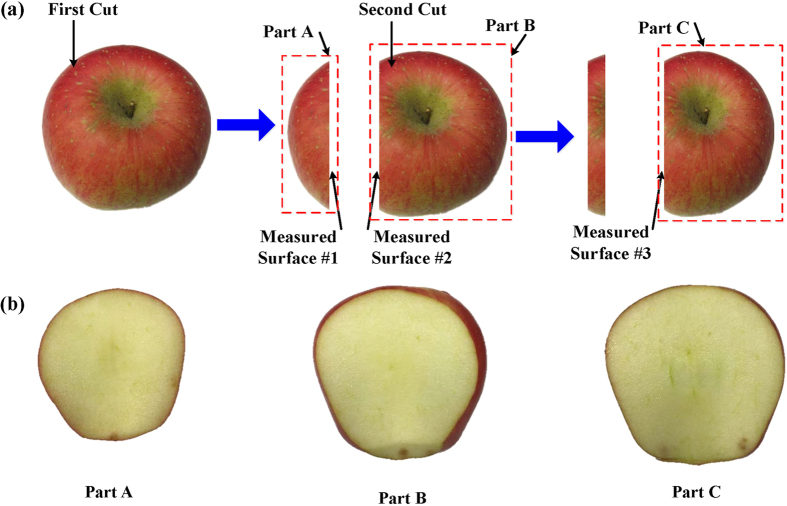
Sample preparation process for individual apple in the SFDI system. (**a**) Sample handling; (**b**) Cross-section views of part A, part B and part C.

Over 60 apples were purchased at a local fruit supermarket, from which 36 apples were selected as test samples for the SFDI measurement. They were chosen to be as uniform as possible with respect to appearance and physical sizes, and their average weight, diameter and height were 232 ± 14.3 g, 81.7 ± 2.37 mm and 68.3 ± 3.42 mm respectively. A sample size larger than 30 apples should have been adequate for statistical analysis, for instance, 40 apples were selected as samples for quality detection and assessment in several studies^23^,^24^. The intact apple samples were cut lengthwise to generate two different measured surfaces (#1, #2), and part A had thickness of approximately 25 mm. A slice (about 10 mm thick) was removed from part B, revealing measured surface #3, roughly 5 mm above the apple core (Fig. 7). The true thickness of each part was recorded as an average across three measurements using digital calipers. The samples were sliced to be flat as possible, placed on the platform, and mounted by the sample holders for SFDI measurement. Since the slicing process was completed manually, slice thickness consistency between the first and second cut could not be guaranteed.

### Optical Measurement

The SFDI measurements were carried out on each of the three measured surfaces at three wavelengths (460, 527, and 630 nm) per sample. These wavelengths are commonly used for apple quality assessment: 460 nm is near an absorption peak corresponding to combination of chlorophyll-a and carotenoids, and the μ_*a*_ is about 5–10 times of that at other wavelengths^19^,^20^; a small absorption peak is expected to be observed at 527 nm due to absorption by anthocyanin in the apple fruit tissue^19^,^33^; the μ_*a*_ at 630 nm is close to the absorption peak of chlorophyll and it is linked to apple fruit maturity, while the 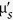 is related to fruit texture^34^. At each wavelength, ten spatial frequencies were selected between 0.01 mm^−1^ and 0.1 mm^−1^ at an interval of 0.01 mm^−1^, corresponding to a total of 93 (31 × 3) images for the three measured surfaces per apple sample. Each image was captured twice, and the two images were averaged in data processing to reduce error in the measurements. Diffuse reflectance images at each spatial frequency were demodulated using equations (5) and (6). The μ_*a*_ and 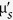 were obtained by fitting the diffuse reflectance images with equation (7). The time required to process the data depended on the size of the selected region of interest (ROI) and wavelength number. In this study, the ROI was chosen as 200 × 300 pixels (about 30 × 45 mm^2^), and 5 × 5 pixel binning was performed on each image to reduce computation time, resulting in 40 × 60 pixel, modulation, diffuse reflectance images.

## Additional Information

**How to cite this article**: Hu, D. *et al*. Noncontact and Wide-Field Characterization of the Absorption and Scattering Properties of Apple Fruit Using Spatial-Frequency Domain Imaging. *Sci. Rep.* **6**, 37920; doi: 10.1038/srep37920 (2016).

**Publisher's note:** Springer Nature remains neutral with regard to jurisdictional claims in published maps and institutional affiliations.

## Figures and Tables

**Table 1:** Calibration results of system accuracy.

Optical property	μ_*a*_ (mm^−1^)			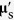 (mm^−1^)		
Wavelength (nm)	460	527	630	460	527	630
Measured value (mm^−1^)	0.0073	0.0050	0.0043	1.077	1.024	0.974
	0.0078	0.0047	0.0035	1.042	0.958	0.952
	0.0072	0.0053	0.0048	0.991	0.976	0.948
	0.0063	0.0051	0.0047	1.012	0.938	0.970
	0.0054	0.0050	0.0046	1.017	0.976	1.002
Average value (mm^−1^)	0.00679	0.00502	0.00438	1.027	0.974	0.969
True value (mm^−1^)	0.00602	0.00472	0.00387	0.954	0.942	0.922
Relative error (%)	12.79	6.36	13.18	7.65	3.40	5.10

**Table 2:** The μ_*a*_ and 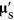 of 36 ‘Redstar’ apple.

Test sample	Measured surface	460 nm		527 nm		630 nm	
		μ_*a*_ (mm^−1^)	(mm^−1^)	μ_*a*_ (mm^−1^)	(mm^−1^)	μ_*a*_ (mm^−1^)	(mm^−1^)
Healthy apple	Measured surface #1	0.0656 ± 0.0099	0.6318 ± 0.0480	0.0173 ± 0.0041	0.6734 ± 0.0480	0.0058 ± 0.0011	0.6703 ± 0.0368
	Measured surface #2	0.0841 ± 0.0162	0.4564 ± 0.0929	0.0212 ± 0.0048	0.5446 ± 0.0620	0.0067 ± 0.0020	0.5966 ± 0.0573
	Measured surface #3	0.0755 ± 0.0152	0.4679 ± 0.1009	0.0234 ± 0.0069	0.5493 ± 0.0796	0.0075 ± 0.0039	0.6198 ± 0.0675
IB apple	Measured surface #1	0.2326	0.6025	0.1323	0.7393	0.1250	0.8563
	Measured surface #2	0.2474	0.4333	0.1633	0.5974	0.1954	0.9101
	Measured surface #3	0.2130	0.3889	0.1535	0.4902	0.1599	0.5555
